# Portal Vein Thrombosis With Hypoplasia in the Left Lobe of the Liver: A Case Report

**DOI:** 10.7759/cureus.52964

**Published:** 2024-01-25

**Authors:** Yosuke Fukuda, Naruhito Oda, Hironori Sagara

**Affiliations:** 1 Department of Medicine, Division of Respiratory Medicine and Allergology, Yamanashi Red Cross Hospital, Fujikawaguchiko-machi, JPN; 2 Department of Medicine, Division of Respiratory Medicine and Allergology, Showa University School of Medicine, Tokyo, JPN

**Keywords:** rivaroxaban, right heart failure, portal vein thrombosis, hypoplasia of the liver, computer tomography

## Abstract

Portal vein thrombosis (PVT) is an acute-onset, emergent thrombotic disease that is difficult to diagnose without an apparent underlying disease unless the clinician actively suspects its presence. We present a case of acute PVT with underlying left lobe hypoplasia of the liver, a previously undescribed condition. A 79-year-old male patient presented to the emergency department with the chief complaint of anorexia. His medical history included hypertension and an old brain infarction. The patient had no history of surgery. Contrast-enhanced CT revealed the disappearance of the left lobe of the liver and defects in the contrast effect in the left portal vein. The diagnosis reached was PVT with left lobe hypoplasia of the liver. Hypoplasia of the liver manifests few symptoms and may be identified incidentally. Clinicians need to be aware that PVT can develop from various underlying conditions, including hypoplasia of the liver, and we recommend aggressive imaging studies to help detect the presence of PVT when encountering similar cases.

## Introduction

Portal vein thrombosis (PVT) affects the hemodynamics of the portal vein and is one of the most severe diseases requiring urgent attention in cases of acute onset. A previous study of patients with virus-related cirrhosis followed up for 11 years reported that 28% developed PVT [1]. In another study involving 3685 patients with liver disease, PVT was identified in 4.2% of patients with cirrhosis and 6.1% of patients with portal hypertension [2]. Although color Doppler ultrasound and contrast-enhanced CT are the gold standard methods for diagnosis [3], it is difficult to suspect the presence of PVT and make an accurate diagnosis in patients with no known preexisting liver disease. We discuss a case of acute PVT with underlying left lobe hypoplasia of the liver, a previously undescribed condition. This report indicates that hypoplasia of the left lobe of the liver is a risk factor for PVT and provides a clue to the proactive identification of PVT.

## Case presentation

**Figure 1:**
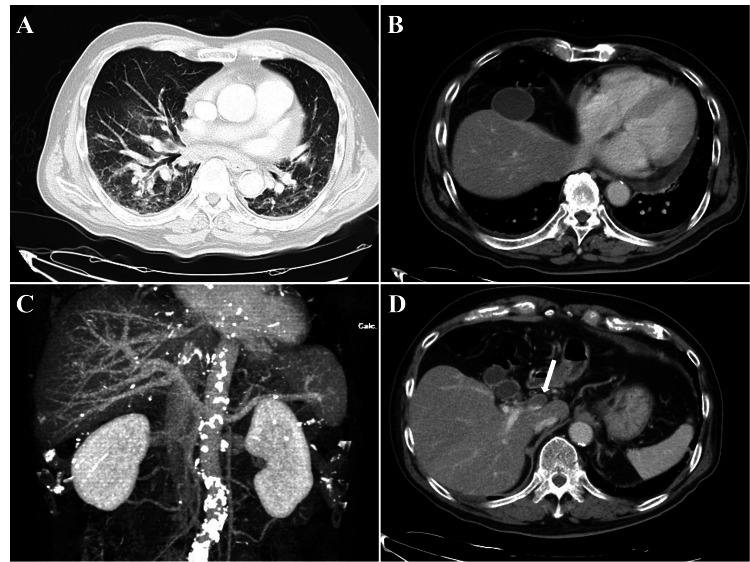
Contrast-enhanced CT on initial presentation (A) Chest CT reveals frosted shadows, and thickened interlobular septal walls in the lung bases bilaterally. (B, C) Abdominal CT reveals the disappearance of the left lobe of the liver and (D) defect of contrast effect in the left portal vein on admission (white arrow) CT: computed tomography

A 79-year-old male patient presented to the emergency department with the chief complaint of anorexia for seven days. His medical history included hypertension and an old brain infarction. The patient had no history of surgery. The patient denied any drug or food allergies. His vital signs during the visit were as follows - unclear consciousness (Glasgow Coma Scale: E4V5M5), body temperature: 36.3 °C, pulse rate: 105 beats per minute, blood pressure: 99/62 mmHg, respiratory rate: 20 breaths per minute, and oxygen saturation: 90% on ambient air. Physical examination revealed holo-inspiratory crackles on the dorsal lung bases bilaterally, and blood examinations showed elevated inflammatory marker (C-reactive protein: 15.92 mg/dL) and liver function tests (aspartate aminotransferase: 58 IU/L, alanine transaminase: 84 U/L, γ-glutamyltranspeptidase: 280 IU/L, and alkaline phosphatase: 62 IU/L), and fibrin/fibrinogen degradation products at 47.2 μg/mL. Blood culture results were negative. Given the possibility of sepsis, we performed contrast-enhanced CT from the neck to the pelvis. It revealed frosted shadows, thickened interlobular septal walls in the lung bases bilaterally (Figure 1A), disappearance of the left lobe of the liver (Figures 1B, 1C), and defects in the contrast effect in the left portal vein (Figure 1D).

**Figure 2:**
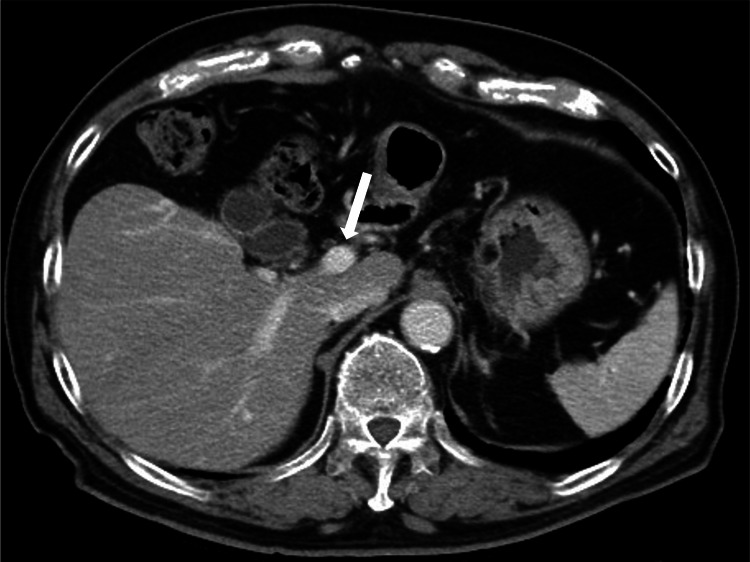
Contrast-enhanced CT imaging after 6 months Portal vein thrombosis disappeared after 6 months of treatment with rivaroxaban (white arrow) CT: computed tomography

The patient was diagnosed with PVT associated with hypoplasia of the left lobe of the liver and acute right-sided heart failure. Rivaroxaban was initiated as an antiplatelet agent, and two months after treatment induction, the filling defect in the portal vein disappeared (Figure 2).

## Discussion

Risk factors for PVT include cirrhosis, malignancy, thrombophilia, history of abdominal surgery, inflammatory abdominal disease including infection, tuberculous lymphadenitis, and abdominal trauma [4]. No risk factors for PVT were documented in our case, and imaging findings were not suggestive of idiopathic portal hypertension [5]. Although the mechanisms of hypoplasia or aplasia of the left or right lobes of the liver have not yet been fully elucidated, previous reports have shown that specific bone morphogenetic proteins from the septum transversum mesenchyme and fibroblast growth factors produced by cardiac mesoderm are essential for liver formation [6,7]. In this case, the left branch of the portal vein had a contrast effect, suggesting that the left lobe of the liver was hypoplastic rather than aplastic, and we speculated that this hypoplasia increased intrahepatic portal venous pressure leading to thrombus formation in the portal vein. 

Merrill reported only one case of a left lobe defect of the liver among 19,000 autopsy cases [8]; however, with the recent advancements in imaging technology, an increasing number of cases are being identified incidentally. In our case, no previous evidence of left lobe hypoplasia of the liver was observed. Hilar bile duct carcinoma is a common cause of acquired liver hypoplasia, and aggressive CT or MRI with contrast is essential to identify it [9]. Indeed, it has been suggested that differences in underlying disease may define the prognosis of PVT, and hence clinicians should actively pursue aggressive imaging studies when PVT is suspected [10]. In addition to anatomical identification of hepatic hypoplasia or aplasia, we believe that accurate imaging evaluation of PVT is important to reduce complications when performing liver transplantation or abdominal surgery [11,12,13].

Due to difficult peripheral intravenous access, the patient was initially treated with rivaroxaban, a direct oral anticoagulant (DOAC) [13-15]. Studies have shown that DOACs containing rivaroxaban are useful for PVT with adequate safety. Although a previous study has shown that rivaroxaban and dabigatran are equally effective for acute PVT in cirrhosis [16], the evidence is not sufficient to recommend these drugs. In addition, the aforementioned reviews also varied in duration of treatment with DOACs, ranging from 1 to 13 months. Therefore, further studies are needed to gain more insights into drug selection and duration of treatment. Chronic thrombi are known to be challenging to recanalize [17]; however, in this case, we considered the patient to have acute-onset PVT because the thrombus had recanalized six months after treatment initiation.

This report has several limitations. Firstly, the patient had not undergone an abdominal ultrasound previously, and it is not clear whether the hepatic left lobe hypoplasia was congenital or acquired. Secondly, although the patient was treated with rivaroxaban for six months, the follow-up has not yet been completed, and further observation is needed to determine whether there are any long-term effects of the treatment. Based on these considerations, we believe careful further follow-up is necessary in this case.

## Conclusions

We presented a rare report describing an uncommon case of PVT with underlying left-lobe hypoplasia of the liver. PVT was resolved with rivaroxaban, and we achieved a good outcome for our patient. PVT can develop in the setting of a variety of diseases. Liver hypoplasia is rarely the underlying cause, and aggressive imaging studies, including contrast-enhanced CT, are essential for diagnosis, using nonspecific symptoms as a clue. Clinicians should be aware that liver hypoplasia can cause PVT. However, there is room for further investigations into the mechanism and epidemiology of this rare entity with an accumulation of cases.
